# Large Language Models and Their Applications in Mental Health: Scoping Review

**DOI:** 10.2196/88057

**Published:** 2026-05-15

**Authors:** Matheus Calvin Lokadjaja, Jordon Junyang Kho, Peter Johannes Schulz, Wilson Wen Bin Goh

**Affiliations:** 1 Lee Kong Chian School of Medicine Nanyang Technological University Singapore Singapore; 2 Wee Kim Wee School of Communication and Information Nanyang Technological University Singapore Singapore; 3 Department of Communication & Media Ewha Womans University Seoul Republic of Korea; 4 Center of AI in Medicine Nanyang Technological University Singapore Singapore; 5 Center for Biomedical Informatics Nanyang Technological University Singapore Singapore; 6 School of Biological Sciences Nanyang Technological University Singapore Singapore; 7 Division of Neurology, Department of Brain Sciences Faculty of Medicine Imperial College London London, England United Kingdom; 8 Institute of Mental Health Singapore Singapore

**Keywords:** artificial intelligence, generative AI, large language models, mental health, natural language processing

## Abstract

**Background:**

Large language models (LLMs) are poised to transform mental health care, offering advanced capabilities in diagnosis, prognosis, and decision support. Since their inception, numerous mental health-focused LLMs have emerged in the scientific literature, reflecting the growing interest in leveraging these models across various clinical applications. With a broad range of models available, diverse optimization strategies, and multiple use cases, reviewing the current landscape is critical to understanding where future impact lies.

**Objective:**

This study aimed to conduct a scoping review investigating the use of LLMs in mental health across diagnostic, prognostic, and decision support tasks.

**Methods:**

We screened 3121 papers from PubMed, Scopus, and Web of Science for studies published between January 2023 and October 2025, using terms related to LLM and mental health. After removing duplicates, 2 reviewers (MCL and WWBG) independently screened the studies, with a third (JJK) to resolve conflicting opinions. We extracted and synthesized information on the models, use cases, datasets, and adaptation methods from selected papers.

**Results:**

In total, 41 papers were selected. Many studies included evaluations on OpenAI’s GPT series applications: GPT-4 (24 studies, 58.5%) and GPT-3.5 (16 studies, 39%). Others included Bidirectional Encoder Representations from Transformers-derived models (9 studies, 22%), LLaMA (8 studies, 19.5%), and RoBERTa-derived models (6 studies, 14.6%). While all studies initially applied out-of-the-box LLMs, several adapted them through few-shot learning or fine-tuning to better align with specific research goals. The most common use case was in diagnostics (31 studies, 75.6%), while the most common target condition was depression (11 studies, 26.8%). While many studies reported superior performance of LLMs, only a minority of studies (13 studies, 31.7%) validated LLM performance against clinician assessments using real patient data, with the majority relying on proxy outcomes such as clinical vignettes, examination questions, or social media posts.

**Conclusions:**

Despite rapid growth and diversity of LLM applications in mental health, the field remains nascent and exploratory. Future developments must emphasize consistent model adaptation procedures to ensure safety and clinical workflow alignment. Models must also be evaluated on robust evaluation criteria by using standardized protocols and real clinical outcome measures.

## Introduction

The advent of large language models (LLMs) represents a transformative shift in mental health care, offering novel opportunities for diagnosis, prognosis, and decision support. Since the inception of ChatGPT (OpenAI) in November 2022, the field has witnessed rapid advancements with the emergence of specialized, fine-tuned models tailored for health care [1,2]. These developments have sparked significant interest in leveraging LLMs to address longstanding challenges in mental health, such as improving access to care, enhancing diagnostic precision, and personalizing treatment strategies [3-7].

Early implementations of general-purpose LLMs demonstrated promising potential in mental health-related tasks, including conversational support and preliminary assessments [8-10]. Riding on this momentum, researchers have developed domain-specific applications such as MentalBERT, MentaLLaMA, and Mental-LLM, which are fine-tuned from out-of-the-box models using mental health-specific datasets, social media posts, and therapeutic dialogues [11-13]. These efforts aim to inject expert clinical knowledge into LLMs, enabling more nuanced and accurate responses tailored to psychiatric contexts. Additionally, innovative techniques like instruction fine-tuning and chain-of-empathy prompting have been introduced to enhance the reasoning and interaction capabilities of these models, especially in complex emotional and diagnostic scenarios [14-16].

Similar to other clinical domains, the potential applications of LLMs in mental health are vast and varied. These include assisting clinicians with diagnostic reasoning, predicting disease progression, and even providing direct patient-facing services such as emotional support or psychoeducation [17-19]. Furthermore, LLMs could streamline administrative processes in mental health settings by summarizing therapy sessions or generating treatment plans based on evidence-based practices [18,20]. These capabilities hold promise for addressing barriers to mental health care access, particularly for underserved populations [4,21,22].

Despite these advancements, a significant translational gap exists between research and real-world clinical deployment. Many LLMs are tested on simulated scenarios, vignettes, medical exams, and social media-derived datasets, raising concerns about their reliability and validity in real-world contexts (eg, a model trained on cleaned, idealized data may not function on real-world data, which may contain noise and biases). Furthermore, the implementation pathway and concomitant ethical implications of deploying LLMs in high-stakes environments like psychiatry warrant additional care informed by clear research evidence. Issues such as data privacy, bias in model outputs, and the risk of misinterpretation must be encapsulated into model development, training, and implementation processes. It is unclear how far the field has progressed while accounting for these critical considerations.

In this scoping review, we assess recent technical developments and the real-world applicability of LLMs in mental health care. We examined model types, tuning methodologies, use case diversity, and evaluation criteria to uncover key trends and highlight approaches that address practical deployment challenges. By identifying where progress is being made, we clarify the fruits of current efforts while also highlighting unresolved conflicts and gaps. We hope this can help AI developers think more critically about how to create more clinically meaningful and ethically sound applications. Simultaneously, applied clinical researchers can use this to raise awareness of various conflicts and synergies based on use case, to help them prioritize deployment testing areas and to implement critical checks, ensuring patient safety and outcomes are prioritized.

## Methods

### Literature Extraction

We conducted literature extraction on PubMed, Web of Science, and Scopus by using a comprehensive battery of search terms on November 4, 2025. To avoid duplication efforts, one author (MCL) performed the initial literature extraction following search term consensus among all authors.

The search strategy used a comprehensive set of keywords related to psychiatry and mental health, in combination with terms related to large language models (LLMs). Psychiatry-related terms included “mental health” and “psychiatry,” while LLM-related terms included “large language model” and “LLM.” Specific model names, such as “Gemini,” “GPT,” “Llama,” “Claude,” and “Deepseek,” were added to broaden and refine the search results. Search terms were combined using Boolean operators (AND/OR) and are applied to titles, abstracts, and topic and keyword fields. Further details on the specific search strategies and a full list of search terms are shown in Multimedia Appendix 1.

Studies were eligible if their first online publication date fell between January 1, 2021, and October 31, 2025. For articles published “online ahead of print,” the electronic publication (Epub) date indexed in the database was used to determine eligibility rather than the later print issue date. This approach ensured consistent application of the predefined search window and avoided exclusion of studies that were available online within the eligibility period but assigned to a later print issue. One article [23], published online ahead of print on July 10, 2025, was identified during citation checking following the primary search and met the inclusion criteria.

We include peer-reviewed, nonreview articles (including online-ahead-of-print) published in English without forward and backward citation search. Preprints and unpublished manuscripts were excluded due to a lack of peer-review quality assurance. To maintain focus on real-world applications, we excluded studies that used or generated synthetic data, given the limited confidence in synthetic data within medical research. We also excluded studies where relevance to psychiatry or mental health was peripheral (eg, studies in ethical AI where mental health was only mentioned in passing). Eligible studies must explicitly state that they cover both LLMs and psychiatry or mental health applications.

### Included Studies

Two independent authors (WWBG and MCL) screened the studies. When there is a difference in opinion, a third independent author (JJK) helped to resolve the difference. A fourth author (PS) acted as an independent methodological assessor to validate processes and workflows. From search parameters, we obtained a corpus of 3121 papers derived from PubMed, Scopus, and Web of Science.

From these 3121 papers, 993 were eliminated due to duplicates. To focus the review on higher-impact and well-indexed outlets, we excluded 1601 papers published in journals not ranked within the top quartile of their respective subject categories according to our university’s journal tier list, which was compiled using bibliometric data from Journal Citation Reports and Scopus (via SCImago Journal Rank). This step was taken to ensure a focus on studies meeting established bibliometric quality thresholds, rather than as a proxy for methodological rigor. Of the remaining 527 papers, 486 were removed because they do not meet the inclusion criteria. We identified 41 studies [12,17,19,20,23-59] of interest (Table 1 for summary of articles).

**Table 1:** Summary of the included studies.

Author	Year	Use case	Target condition	Dataset type	Modalities	LLM^a^ used	LLM type	Fine-tuning status	Knowledge augmentation	Country
Schubert et al [24]	2023	Diagnosis	General mental health	Medical examination	Text	GPT-4; GPT-3.5	Decoder-only	Untuned	—^b^	Germany
Watari et al [28]	2023	Diagnosis	General mental health	Medical examination	Text	GPT-4	Decoder-only	Untuned	—	Japan
Rojas et al [25]	2024	Diagnosis	General mental health	Medical examination	Text; Visual	GPT-3.5; GPT-4; GPT-4 with Vision	Decoder-only	Untuned	—	USA
Li et al [26]	2024	Diagnosis	General mental health	Medical examination	Text	GPT-4; Bard; LLaMA-2	Decoder-only	Untuned	—	Taiwan
Herrmann-Werner et al [29]	2024	Diagnosis	General mental health	Medical examination	Text	GPT-4	Decoder-only	Untuned	—	Germany
Kim et al [27]	2025	Diagnosis	General mental health	Medical examination	Text; Visual	Claude 3.5 Sonnet; Gemini 1.5 Pro; GPT-4o	Decoder-only	Untuned	—	South Korea
Levkovich and Elyoseph [32]	2023	Diagnosis	Suicide	Vignettes	Text	GPT-4; GPT-3.5	Decoder-only	Untuned	—	Israel
Levkovich and Elyoseph [17]	2023	Diagnosis	Depression	Vignettes	Text	GPT-3.5; GPT-4	Decoder-only	Untuned	—	Israel
Gargari et al [31]	2024	Diagnosis	Suicide	Vignettes	Text	Aya, GPT-3.5, GPT-4, GPT-3.5 Clinical Assistant (CA), Nemotron, and Nemotron CA	Decoder-only	Untuned	RAG^c^	Iran
Kim et al [33]	2024	Diagnosis	Obsessive-compulsive disorder	Vignettes	Text	GPT-4; LLaMA-3; Gemini-Pro	Decoder-only	Untuned	—	USA
Choi et al [35]	2024	Diagnosis	Delirium	Vignettes	Text	GPT-3.5; GPT-4	Decoder-only	Untuned	—	USA
Wislocki et al [34]	2025	Diagnosis	Trauma	Vignettes	Text	Gemini 1.5 Flash; GPT-4o mini, Claude Sonnet; LLama 3	Decoder-only	Untuned	—	USA
Ohse et al [36]	2024	Diagnosis	Depression	Clinical dataset	Text	GPT-4; GPT3.5; Llama2-13B; BERT	Encoder-only; Decoder-only	Fine-tuned; Untuned	—	Germany
Ghosh et al [43]	2024	Diagnosis	Depression	Clinical dataset	Text; Audio; Visual	BERT	Encoder-only	Fine-tuned; Untuned	—	Australia
Sadeghi et al [50]	2024	Diagnosis	Depression	Clinical dataset	Text; Audio; Visual	GPT-3.5; DepRoBERTa	Encoder-only; Decoder-only	Fine-tuned; Untuned	—	Germany
Arslan et al [52]	2024	Diagnosis	Schizophrenia-spectrum disorders	Clinical dataset	Text	SBERT	Encoder-only	Untuned	—	Turkey
Shi et al [38]	2025	Diagnosis	Obsessive-compulsive disorder; Trauma	Clinical dataset	Text	Mental-LLaMa; MentalQLM; GPT-4	Decoder-only	Fine-tuned; Untuned	—	China
Palominos et al [49]	2025	Diagnosis	Schizophrenia	Clinical dataset	Text	BERT	Encoder-only	Untuned	—	Spain
Leng et al [41]	2025	Diagnosis	Cognitive impairment	Clinical dataset	Text	GPT-4o-mini	Decoder-only	Fine-tuned; Untuned	—	USA
Shin et al [37]	2024	Diagnosis	Depression; suicide risk	Personal data	Text	GPT-3.5; GPT-4	Decoder-only	Untuned	—	South Korea
van Buchem et al [40]	2024	Diagnosis	Depression	Personal data	Text	BERT; RedditBERT	Encoder-only	Untuned	—	Netherlands
Bartal et al [51]	2024	Diagnosis	Posttraumatic stress disorder	Personal data	Text	GPT-3.5	Decoder-only	Fine-tuned; Untuned	—	USA
Thomas et al [46]	2025	Diagnosis	Suicide ideation and advanced suicidal engagement	Personal data	Text	XLM-RoBERTa-base	Encoder-only	Untuned	—	Switzerland
Chung et al [39]	2025	Diagnosis	Depression	Personal data	Text	BERT; BERTopic	Encoder-only; Decoder-only	Untuned	—	South Korea
Xu et al [12]	2024	Diagnosis	General mental health	Social media	Text	Mental-Alpaca; Mental-RoBERTa; Mental-Flan-T5; Flan-T5; Alpaca; BERT; Llama-2; GPT-3.5; GPT-4	Encoder-only; Decoder-only	Fine-tuned; Untuned	—	USA
Dalal et al [48]	2024	Diagnosis	Depression	Social media	Text	LongFormer; RoBERTa; BERT; ERNIEv2; MentalBERT; PsychBERT; ClinicalT5; MentalT5; MentalBART; MentaLLAMA	Encoder-only; Decoder-only	Fine-tuned; Untuned	—	India
Bouktif et al [47]	2025	Diagnosis	Suicide ideation	Social media	Text	BERT	Encoder-only	Untuned	—	UAE
Esmi et al [42]	2025	Diagnosis	Stress	Social media	Text	GPT-4	Decoder-only	Untuned	—	Netherlands
Kallstenius et al [45]	2025	Diagnosis	General mental health	Social media	Text	GPT-4o-mini	Decoder-only	Fine-tuned; Untuned	—	Sweden
Elyoseph et al [53]	2024	Prognosis	Depression	Vignettes	Text	GPT-3.5; GPT-4; Bard; Claude	Decoder-only	Untuned	—	Israel
Elyosep and Levkovich [54]	2024	Prognosis	Schizophrenia	Vignettes	Text	GPT-3.5;GPT-4; Bard; Claude	Decoder-only	Untuned	—	Israel
Lee et al [19]	2024	Prognosis	Suicide ideation	Personal data	Text	GPT-4	Decoder-only	Untuned	—	USA
Perlis et al [59]	2024	Decision Support	Bipolar depression	Vignettes	Text	GPT-4 Turbo	Decoder-only	Untuned	RAG	USA
Adhikary et al [20]	2024	Decision support	General mental health	Clinical dataset	Text	BART; T5; GPT-2; GPT-Neo; GPT-J; Flan-T5; Mistral; MentalBART; MentalLlama; Llama-2; Phi-2	Encoder-only; Decoder-only; Encoder-Decoder	Fine-tuned; Untuned	—	India
So et al [55]	2024	Decision Support	Posttraumatic stress disorder	Clinical dataset	Text	GPT-3.5 Turbo; GPT-4 Turbo	Decoder-only	Fine-tuned; Untuned	—	South Korea
Taylor et al [58]	2024	Decision Support	General mental health	Clinical dataset	Text	RoBERTa-base; RoBERTA-base-OHFT; Clinical Longformer	Encoder-only; Decoder-only	Fine-tuned; Untuned	—	UK
Mahbub et al [56]	2025	Decision support	Substance use disorder	Clinical dataset	Text	Flan-T5	Encoder-Decoder	Untuned	—	USA
Chen et al [23]	2025	Decision Support	General mental health	Clinical dataset	Text	Deepseek R1 Dis-Qwen; Internlm2.5; opt model; gpt-sw3 model; Qwen model	Decoder-only	Fine-tuned; Untuned	—	China
Liu et al [57]	2025	Decision Support	Schizophrenia	Clinical dataset	Text; Audio; Visual	Claude 3 Haiku; Gemini 1.0 Pro; GPT-3.5 Turbo	Decoder-only	Untuned	—	Taiwan
D'Souza et al [30]	2023	Diagnosis; Prognosis	Psychiatry	Vignettes	Text	GPT-3.5	Decoder-only	Untuned	—	India
Abdullah and Negied [44]	2024	Diagnosis; Prognosis	ADHD; anxiety; bipolar; depression	Social media	Text	BERT; RoBERTa; OpenAI GPT; GPT 2	Encoder-only; Decoder-only	Untuned	—	Egypt

^a^LLM: large language model.

^b^Not available.

^c^RAG: retrieval-augmented generation.

### Use Case Stratification

Selected studies were divided into three main use cases based on explicit examination of their objectives: diagnosis, prognosis, and decision support. Diagnostic studies leverage LLM capabilities to detect mental health conditions directly from text and evaluate diagnostic performance. Prognostic studies focus on predicting future mental health outcomes with LLMs. Decision support studies examine various LLM-based approaches to assist clinicians in making informed decisions about patient care, such as providing advice on treatment protocols and evidence search.

Each study was assigned to one or more use-case categories. Studies addressing multiple use cases with comparable emphasis (eg, both diagnostic classification and prognostic prediction) were assigned to each relevant category. These studies will be discussed in their respective subsections to ensure their contribution to each use case are fully captured in the analysis. Consequently, studies were intentionally counted in all applicable categories, and percentage calculations were based on the total number of included studies (N=41) [12,17,19,20,23-59], with the understanding that category percentages are not mutually exclusive and may exceed 100% when summed. Coding decisions were conducted independently by 2 reviewers (MCL and WWBG), with disagreements resolved through discussion until consensus was reached.

### Data Extraction

For each study, 2 authors (WWBG and MCL) performed full-text extraction and coded the articles. We extracted the following information from the full-text: study information (author’s name, year of publication, and country of study), LLM use case, what their role is, the target condition, type of dataset, and the modalities, LLM type and configuration, tuning status, and presence of knowledge augmentation. As there were many types of datasets across studies, we defined five types of datasets: medical examinations, vignettes, clinical datasets, personal data, and social media posts. During the extraction process, when there was a difference in opinion, a third independent author (JJK) helped resolve the difference. To ensure protocol was strictly adhered to and in line with best practices, we reviewed processes with an independent senior author (PS).

Medical examination data were defined as standardized examination materials that include an explicit psychiatric or mental health component. While most studies in this category did not focus exclusively on actual mental health applications, they were included if the evaluated task involved mental health–relevant reasoning or assessment. Given the limited number of studies examining direct LLM applications in mental health, inclusion of such studies aligns with the objective of a scoping review to map the breadth of existing evidence. Moreover, the inclusion of these studies enables comparison of LLM performance across different medical subfields.

Vignette data are compiled from case narratives presenting clinical scenarios on mental health conditions. Clinical datasets contain real-world patient data recorded by health care professionals (eg, electronic health records [EHRs] and clinical notes). Personal data are directly collected from individuals (eg, diary entries and messages). Social media posts are sourced from social media platforms (eg, Reddit and Facebook). We categorized the LLM tuning status into untuned and fine-tuned. Untuned LLMs are out-of-the-box models not trained in any specific domain. Fine-tuned LLMs are models that have been trained for a mental health-specific task. The country of study was recorded as the country of the corresponding author.

## Results

### Executive Summary

Broadly, most studies used applications based on OpenAI’s GPT series: GPT-4 (n=24, 58.5%) [12,17,19, 24-29,31-38,41,42,45,53-55,59] and GPT-3.5 (n=16, 39%) [12,17,24,25,30-32,35-37,50,51,53-55,57]. Other popular models include Google’s Bidirectional Encoder Representations from Transformers (BERT) and its derivatives (n=9, 22%) [12,36,39,40,43,44,47-49], META’s Llama series (n=8, 19.5%) [12,20,26,33,34,36,38,48], and RoBERTa and its derivatives (n=6, 14.6%) [12,44,46,48,50,58]. The most used LLM type is decoder-only model (n=34, 82.9%) [12,17,19,20,23-39, 41,42,44,45,48,50,51,53-55,57-59], followed by encoder-only model (n=14, 34.1%) [12,20,36,39,40,43,44,46-50,52,58].

**Figure 1:**
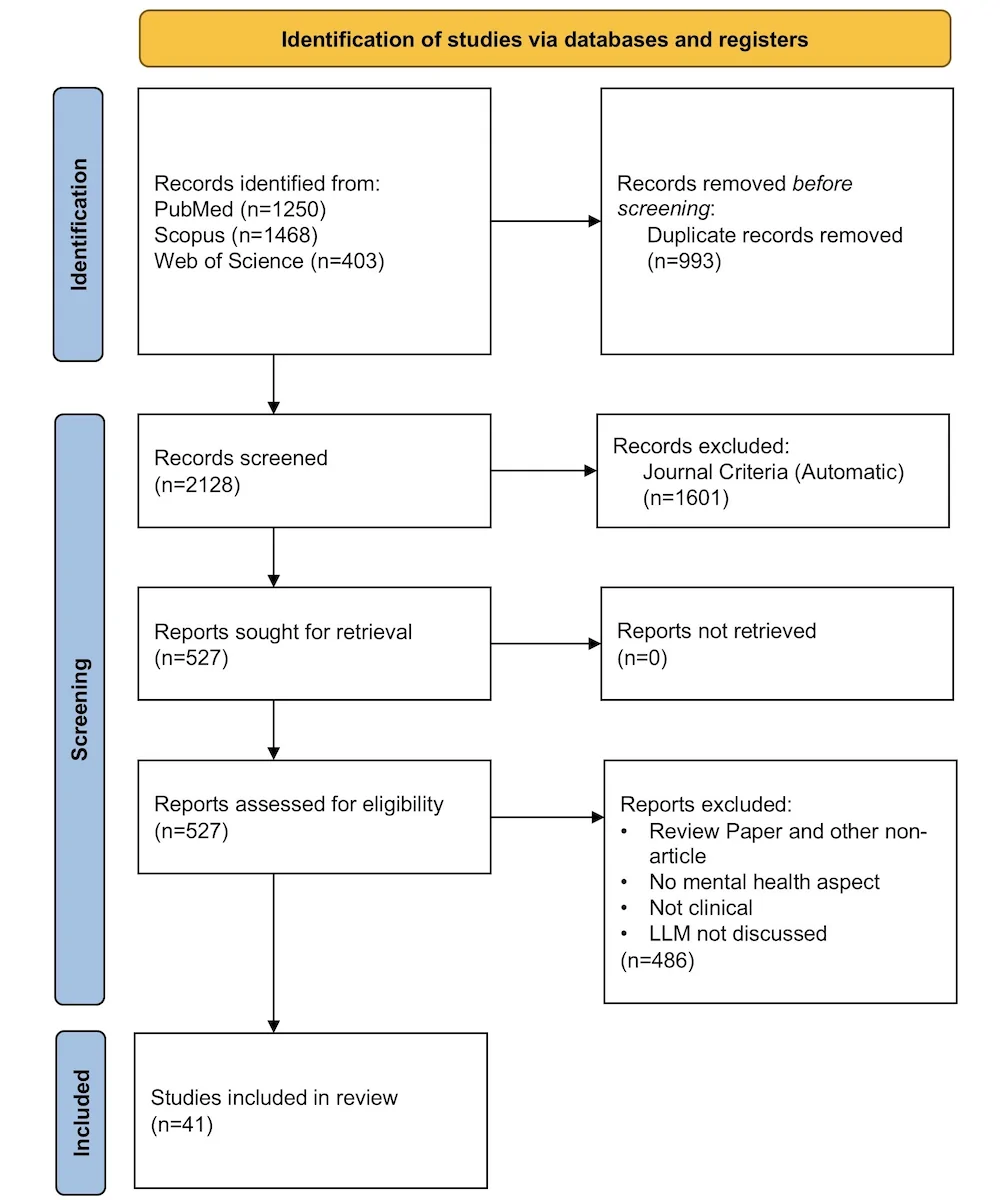
Schematic diagram of the studies in this review.

All studies used out-of-the-box models with about a third also evaluating domain-specific fine-tuned derivatives (n=13, 31.7%) [12,20,23,36,38,41,43,45,48,50,51,55,58]. The most common use-case was diagnosis (n=31, 75.6%) [12,17,24-52], followed by decision support (n=7, 17.1%) [20,23,55-59]. Among mental conditions, the most studied was depression (n=11, 26.8%) [17,36,37,39,40,43,44,48,50,53,59]. Other conditions studied include attention deficit hyperactivity disorder, obsessive-compulsive disorder (OCD), and suicidal ideation (Figure 1).

### Diagnosis Is the Most Common Use Case for LLMs in Mental Health

#### Overview

We divided use cases into three categories: diagnosis, prognosis, and decision support. Diagnosis involves identifying mental health conditions, while prognosis predicts their likely course and outcome. Decision support encompasses tools that help clinicians and patients make informed choices about care. Of the selected studies, most focused on diagnosis where the LLM, usually untuned, was used to diagnose a mental health disorder directly from input (n=31, 75.6%) [12,17,24-52].

#### Diagnostic Evaluations on Medical Examinations

Of 31 relevant studies [12,17,24-52], 6 [24-29] evaluated LLMs via performance in medical exams. These controlled assessments evaluate the degree of knowledge retrieval in out-of-the-box GPT models, with the expectation that a pretrained model, knowledgeable in the clinical domain, has potential for clinical deployment. Model performance has improved markedly across GPT iterations: When Schubert et al [24] compared both GPT-4 and GPT-3.5 to answer neurology board-style exam questions, GPT-4 performance surpassed the performance of GPT-3.5 and humans in the behavioral, cognitive, and psychological categories. This result was also corroborated by Rojas et al [25], who showed that GPT-4 and GPT-4V are superior to GPT-3.5 when taking the Chile’s major medical examination, Examen Único Nacional de Conocimientos de Medicina, for the psychiatric portion, they perform well.

Across multimodel comparisons, GPT consistently outperformed alternative LLMs. Li et al [26] showed that GPT-4 was able to pass the 2022 Taiwan Psychiatric Licensing Examination, whereas Bard and Llama-2 failed. When tested for differential diagnosis, it was reported that GPT-4 performed close to an experienced psychiatrist [26]. Following the trend of testing the ability of LLM to perform in non-English tests, Kim et al [27] showed that GPT-4o, Claude 3.5, and Gemini 1.5 Pro all performed well on taking the Korean medical licensing examination, with GPT-4o and Claude 3.5 outshining the Gemini 1.5 Pro in all categories, including psychiatry.

However, there are also noteworthy limitations, especially when it comes to hallucinations. Schubert et al [24] reported that regardless of GPT version, mistakes and wrong information are presented with complete confidence especially when challenged with higher-order cognitive type problems [24]. When Watari et al [28] compared the performance of GPT-4 and the average Japanese medical resident when taking the Japanese General Medicine In-Training Examination, they found that while GPT-4 outperformed the average medical resident, the medical resident outperformed GPT-4 in the category of “psychiatry” [28]. Furthermore, compared to other clinical categories, GPT-4 performed worst in “psychiatry,” demonstrating the specific domain challenge pertinent to psychiatry and mental health. Moreover, there is also the issue of black boxes and catastrophic forgetting. Herrmann-Werner et al [29] pointed out that although GPT-4 passed the exam with more than 90% accuracy in the evaluation of clinical diagnostics, when GPT-4 was incorrect, the algorithm showed that they were unable to “remember” or “understand” the context of the problem [29]. Similarly, casting doubt on real-world deployment, Li et al [26] also posit that while GPT-4 can pass the Taiwan Psychiatric Licensing Examination, it is also noted that it performs worse compared to experienced psychiatrists. Such unexpected failure can have important implications regarding patient safety, clinical efficacy, and even liabilities in real-world deployment.

#### Diagnostic Evaluations on Vignettes

Vignettes are another important evaluation scenario, with the key difference being that vignettes take the form of unstructured narratives. Seven selected studies evaluated LLMs on clinical vignettes. Here, the LLMs are evaluated for their ability to apply the knowledge that they learned by extracting relevant information from vignettes to form and justify their diagnosis.

Here, LLMs generally display good performance. Franco D'Souza et al [30] used 100 cases of psychiatry vignettes to evaluate GPT-3.5, reporting exceptional performance, especially in forming management strategies and diagnoses from the scenario. Gargari et al [31] also showed that GPT-4, GPT-3.5, and GPT-3.5 with RAG can perform well in diagnostic tasks when presented with 20 clinical vignettes. In some cases, LLMs can perform as well or better than mental health professionals as shown by Levkovich and Elyoseph [32] who compared GPT-3.5 and GPT-4 with mental health professionals in the case of clinical vignettes related to suicide, and by Kim et al [33] who compared GPT-4, LLaMA-3, and Gemini-Pro with mental health professionals in the case of OCD *DSM-5* (*Diagnostic and Statistical Manual of Mental Disorders, Fifth Edition*) clinical casebook, respectively.

Another key advantage was reported by Levkovich and Elyoseph [17], where they evaluated GPT-3.5 and GPT-4 for their evaluation in depression vignette cases and found that they are in line with official guidelines. But more than that, they also found that when compared against physicians, LLMs displayed no biases with regard to the gender or socioeconomic status of the patient [17]. Similarly, Wislocki et al [34] found that the LLMs that they used (Gemini 1.5 Flash, GPT-4o mini, Claude Sonnet, and Meta Llama 3) demonstrated less trauma-related diagnostic overshadowing bias when presented with vignette about OCD and substance abuse symptoms. This is an important result, seeing as issues of model discrimination and inequity are often raised as reasons for non-adoption.

However, there are limitations. In Levkovich and Elyoseph’s study with suicide vignettes, GPT-3.5 tended to underestimate the risk of suicide [32]. In their work with depression vignette cases, both GPT-3.5 and GPT-4 showed difficulty in assessing vignettes containing forbidden content (sexual violence), which violate rules of use. Choi et al [35], while showing that GPT-3.5 and GPT-4 can work well in delirium vignette cases, found that there is difficulty for the models to comprehend complex use cases, especially when tasked to justify their thought processes. Gargari et al [31] also cautioned that the models performed worse with specific disorders such as cyclothymic and disruptive mood dysregulation disorders. They also reported that GPT-4 and GPT-3.5 outperformed AYA and Nemotron models, which may highlight a performance difference between open-source and proprietary models.

#### Diagnostic Evaluations on Clinical Datasets, Personal Data, and Social Media Posts

Unlike medical examinations and vignettes, texts from clinical datasets, personal data, and social media posts tend to be written in a freestyle manner (ie, no specific formatting) and contain nonclinical terms. We found 18 studies [12,36-52] evaluating LLMs on these data types and exploring novel frameworks to enhance diagnostic performance and clinical explainability.

Most studies reported that fine-tuning is crucial for higher diagnostic performance. Ohse et al [36] compared out-of-the-box GPT-4, GPT-3.5, Llama2-13B, and BERT in diagnosing depression from clinical patient interviews. They reported that while GPT-4 was the best-performing untuned model, it did not outperform fine-tuned GPT-3.5. Separately, Shin et al [37] reported that fine-tuned GPT-3.5 performed best in diagnosing depression and suicide risk from diary texts as compared to untuned GPT-4 and GPT-3.5. Similarly, Xu et al [12] and Shi et al [38] demonstrated that fine-tuned LLMs outperformed out-of-the-box LLMs in detecting various mental health conditions from Reddit, Twitter, and other social media posts. Chung et al [39] also found that BERT model using the Patient Health Questionnaire-9 (PHQ-9) as a benchmark can screen clinical depression from text messages with high performance. However, van Buchem et al [40] noted that performance increase from fine-tuning is not always significant. They did not find a significant increase in diagnostic accuracy between BERT and fine-tuned RedditBERT in detecting depression from patient messages to caregivers.

What seemed to work is by adding self-evaluating steps to enhance reliability of the LLM, as shown by Leng et al [41], who made a framework for the classification of stages of cognitive impairment from EHR. Another method to improve LLM performance is by altering the prompt – prompt engineering – as shown by Esmi et al [42] who provide hints in the prompt, which made GPT-4 to surpass domain-specific models (MentalQLM and Mental-RoBERTa) in detecting stress from social media data.

Most studies reported that LLMs achieved higher diagnostic accuracies than traditional machine learning (ML) models when applied to the same task. Van Buchem et al [40] reported that BERT and RedditBERT outperformed logistic regressors and support vector machines (SVMs) in diagnosing depression from patient messages and were able to qualitatively provide explanations. Ghosh et al [43] demonstrated that BERT-based models performed best in diagnosing depression from clinical interviews as compared to random forests, SVMs, convolutional neural networks (CNNs), and long short-term memory (LSTM) networks. Furthermore, Abdullah and Negied found that BERT, RoBERTa, GPT, and GPT-2 outperformed ML models in diagnosing attention deficit hyperactivity disorder, anxiety, bipolar disorder, and depression from Reddit posts in clinical subreddits [44]. However, this notion is challenged by Kallstenius et al [45] who compared GPT-4o, out-of-the-box and fine-tuned, against traditional ML and NLP methods with advanced feature engineering. They found that traditional ML performed better against fine-tuned and the out-of-the-box LLM.

There have also been efforts to merge LLMs and ML models into hybrid models to improve overall diagnostic performance. For instance, Thomas et al [46] developed an LLM-multilayer perceptron (LLM-MLP) model with XLM-RoBERTa-base as an encoder to detect suicide risk from a German crisis helpline dataset, and found that it outperformed word2vector-MLP, a non-LLM hybrid model. Similarly, Bouktif et al [47] reported that combining BERT with CNNs and LSTM networks improved the detection of suicidal ideation from Reddit posts. Besides integration with deep learning models, LLMs have been used as building blocks in more complex frameworks to enhance explainability and diagnostic performance. For example, Dalal et al [48] developed a BERT-based model infused with PHQ-9 lexicon and reported that it outperformed BERT and mental health-specific BERT models in diagnosing depression from Reddit posts. Palominos et al [49] used small LLMs such as BERT and sentence Transformers to make sentence embeddings that are then used to make a single composite index that can reliably classify schizophrenia spectrum disorders and track their symptoms over time.

Several studies have also leveraged LLMs to extract input features for ML diagnostic models. Sadeghi et al [50] used GPT-3.5 and DepRoBERTa to extract depression severity features from E-DAIC interview transcripts. These features were evaluated on SVMs and were able to detect depression with good performance. Bartal et al [51] generated text embeddings from text-embedding-ada-2002 from unstructured written text narratives. They evaluated these embeddings on a neural network model to detect childbirth-related posttraumatic stress disorder and found that the model outperformed GPT-3.5’s zero-shot and few-shot classifications. Going beyond English-language datasets, Arslan et al [52] demonstrated the versatility of sentence-transformer models by using SBERT on speech samples from Turkish-speaking patients to detect schizophrenia-spectrum disorders. By generating embeddings from interview transcripts and applying traditional ML classifiers, they achieved high diagnostic accuracy, showcasing the adaptability of LLM-based approaches across languages and clinical conditions.

However, a common challenge remains when applying LLMs for diagnostic role on unstructured data. Xu et al [12] noted that the LLM struggled with processing complex contextual sentences, especially given the nonspecific nature of social media posts, which may limit generalizability across different population groups. Furthermore, Esmi et al [42] mentioned that there is no established method to systematically evaluate the soundness of the reasoning of the LLM output when prompted. In the same vein, Abdullah and Negied [44] also highlighted that while LLMs outperformed machine learning classifiers on clinical subreddit content, traditional ML classifiers outperformed LLMs when presented with nonclinical subreddit content. This underscores the importance of data context and source in model performance. On a more fundamental level, Ohse et al [36] highlighted that open-source LLMs tend to have an older knowledge cutoff compared to their closed-source counterparts, which may affect their clinical use. In the field of mental health, where privacy and data protection are key concerns, this performance gap between open and closed-sourced models may prove to be an obstacle to widespread adoptability. Additionally, van Buchem et al [40] brought up the presence of bias, which can be seen by the variations in performance across different patient groups, especially with regard to racial and ethnic background.

### Prognosis

Five studies [19,30,44,53,54] covered the use of LLMs in prognosis. These covers scenarios involving using AI to predict likely course and outcome of mental health diseases across a variety of data landscapes, including vignettes, personal data, and social media posts.

In a prognostic role, while generally LLMs performed well, there are nuances. When answering clinical vignettes about prognosis, Franco D'Souza et al [30] reported that GPT-4 achieved high performance, attaining the highest grade for most of the vignettes. This high performance was also reported by Elyoseph et al [53] when they used GPT-4, Bard, and Claude to analyze clinical vignettes about long-term outcome of major depressive disorder and found their predictions were comparable to experts; by Elyoseph and Levkovich [54], again using GPT-4, Bard, and Claude to analyze clinical vignettes related to long-term outcomes of schizophrenia and also found their predictions to be comparable to experts; and by Lee et al [19] using GPT-4 to predict future mental health crisis using telehealth data and showed that GPT-4 performed comparably to expert clinicians. Moreover, GPT-4 can extract relevant risk indicators, which can explain their thought process and build trust for LLM predictions.

However, there are caveats in their findings. Other than GPT-4, Bard, and Claude, Elyoseph et al [53] also evaluated GPT-3.5 and found that its predictions were more pessimistic than experts. Elyoseph and Levkovich [54] similarly evaluated GPT-3.5 and found that its predictions were more pessimistic than experts. Lee et al [19] observed that GPT-4 has a high number of false positives compared to clinicians [19]. GPT-4 also performed particularly well when clinicians showed high agreement in their assessments, suggesting that GPT-4 is stronger in extracting obvious, widely recognized clinical signs but may perform less reliably on more ambiguous or nuanced cases. This argument is supported by the findings of Abdullah and Negied [44] who compared between ML and ensemble learning classifiers and 4 out-of-the-box LLMs: BERT, RoBERTa, OpenAI GPT, and GPT-2 in predicting future mental disorders. They found that ML and ensemble learning classifiers outperformed the LLM models.

### Decision Support

Seven studies [20,23,55-59] covered the use of LLMs in decision support where AI is used to assist in informing decisions about a patient’s care to the clinicians. These applications fall into two main categories: information extraction and summarization, and direct clinical recommendation.

One approach to information extraction involves automatic identification of relevant and critical information from lengthy exchanges between patient and doctor in real time. Adhikary et al [20] evaluated an array of LLMs–BART, T5, GPT-2, GPT-Neo, GPT-J, FLAN T5, Mistral, MentalBart, MentalLlama, Llama 2, and Phi 2-in summarizing mental health counseling sessions which may enable faster decision-making by providing concise, relevant session highlights [20]. They found that domain-specific LLMs (MentalBart and MentalLlama) outperformed general-purpose models. Similarly, when summarizing psychiatric interviews for symptoms delineation, So et al [55] found that domain-specific model GPT-3.5 performed better than general-purpose GPT-4. Even so, with out-of-the-box Flan-T5 model, Mahbub et al [56] demonstrated that Flan-T5 outperformed rule-based regular expressions in extracting key information from substance use disorder clinical notes. This can be attributed to LLMs being able to understand nuanced and diverse expressions in the clinical notes. This ability also extends to non-English transcripts shown in the study by Liu et al [57], which used LLM to evaluate the motivation and pleasure domain of negative symptoms. The LLMs such as Claude 3-Haiku, Gemini-10 Pro, and GPT-3.5 Turbo were used to extract key information from Chinese interview transcripts, provide severity score and reasoning behind it, which can then be used by the clinician to base their decision.

For a direct clinical recommendation approach, Taylor et al [58] fine-tuned multiple BERT-based language models–TinyBERT, MobileBERT, DistilBERT, BERT–in triaging patients from the National Health Service electronic health record (NHS EHR) dataset with general mental health conditions. They found that these fine-tuned models achieved high accuracy and outperformed the larger language models, such as LLama 2-7B, when computational resource is limited. Similarly, Chen et al [23] showed that lightweight LLM is able to consume medical data from electroencephalograms. With the information obtained, the LLM can generate emotional states of the patient and suggest diagnostic and treatment suggestions. Domain-specific model superiority was also reported by Perlis et al [59], where their prompt-augmented GPT-4 outperformed the base model in analyzing bipolar disorder clinical vignettes and coming up with their own recommendation, it even outperformed community clinicians.

However, there are notable concerns. While LLMs reported stellar quantitative performance, Adhikary et al [20] also conducted qualitative assessments and consultations with key stakeholders such as the health care professionals. It was agreed that LLMs are not yet reliable enough for clinical deployment, particularly due to issues where LLMs struggle to distinguish clinical details from therapist interpretations and sometimes miss critical nuances such as suicide risk. This insufficiency highlights that some LLMs currently lack an understanding of emotions, nuances, and subtexts, which are important in real-world conversations. This may partly be attributed to older models being used in their study. In contrast, So et al [55] with a newer model, GPT-3.5, demonstrated that the LLM can be tuned to a high degree of granularity, allowing it to pick out crucial information, and even pinpoint specific utterances associated with psychiatric symptoms. Another persisting concern that was aired is hallucination. Mahbub et al [56] reported LLMs’ tendency to hallucinate when confronting notes with more than one substance use disorder condition with varying severity, as well as their limited context window. Chen et al [23] mentioned that larger language model has a tendency to be less precise than smaller language model in their terminology for specific domain context.

## Discussion

### Principal Findings

Our scoping review identified several studies that show predominantly positive findings regarding LLM performance relative to traditional ML and deep learning approaches, and for some even exceeding mental health professionals. However, these findings should be interpreted cautiously. Publication bias likely skews the literature toward positive results, as studies suggesting otherwise are unlikely to be published in today’s AI-centric age, hence potentially skewing the publication toward positive findings.

Despite this limitation, we do see many interesting implementations and use cases for LLMs that hold potential. In some studies, they used a combination of LLM models, and even combined LLMs with traditional AI and ML models (eg, CNN and LSTM) to achieve even higher performance. This seems to be a powerful approach, and could be explored further, especially with greater technical depth. This was also done to augment the capability of the LLM in processing not only textual data but also other modalities. Most studies (n=37, 90.2%) [12,17,19,20,23,24,26,28-42,44-49,51-56,58,59] focused only on textual content, but in mental health, we should also consider the importance of sentiments and emotions, which can be picked up in other modalities, such as audio and visual. An emotionally aware AI may be more well-suited to pick up aberrations in behaviors, which also enhances mental health detection capabilities. While the benefit of domain-specific fine-tuning has previously been reported in other fields [60-62], only about a third (n=13, 31.7%) [12,20,23,36,38,41,43,45,48,50,51,55,58] used fine-tuned model. This may be because model fine-tuning uses significant computational resources, which may not be readily available to those research teams.

We noted the proliferation of models and use cases, reflecting the inherently complex and heterogeneous nature of both technological and clinical landscapes in mental health AI. However, this diversity also exposes several critical concerns. Notably, there is significant variability in approaches to model fine-tuning and a lack of consistency in the diversity and representation of training data. These disparities may risk perpetuating access inequalities and under-representation, particularly among vulnerable or non-Western populations, which may ultimately impact the generalizability and fairness of these models.

Moreover, the absence of standardized testing and evaluation frameworks further exacerbates these challenges. Inconsistent methodologies can hinder repeatability and reproducibility, undermining trust in model performance and potentially leading to unintended harm to patients. Inconsistent data (from different sources), language, dataset specificity, and confounding can also affect model performance. This fragmentation highlights the urgent need for the development and adoption of standardized protocols for model tuning, testing, and evaluation—spanning the entire lifecycle from initial development to real-world implementation.

Current works still rely too much on simple performance metrics. However, LLM research also needs to investigate how to understand and evaluate LLM explanations, ensuring they are acceptable to clinicians and patients, supporting their potential for real-world clinical decision support. The development of explainable models that can act as a domain-level contrast to LLMs will be useful and provide a new level of much-needed scalability, as it is impractical to keep relying on health care professionals to test-gauge LLM and other AI models, taking away time that was supposed to be saved (in theory) by AI.

Placing patients at the center of this technological transformation requires not only technical rigor but also a commitment to equity, transparency, and safety. Establishing robust, standardized frameworks is essential to ensure that AI-driven tools in mental health are both effective and ethically deployed, ultimately safeguarding patient well-being and promoting broader access to high-quality mental health care. We have not seen research that actively involved patient advocacy and involvement of patient perspectives. Moving beyond the technical realm, and actively engaging patients as the center stakeholder is critical and may even accelerate AI adoption in mental health care institutions. However, this requires institutional and even social mindset changes, which is a big topic unto itself.

We also noted a greater preference for using closed-source LLMs (eg, OpenAI’s GPT) in current studies, possibly due to their superior performance and exposure to larger and more recent datasets as compared to open-source LLMs. However, closed-source LLMs pose an inherent risk of data leakage, which can jeopardize patient privacy and confidentiality. To address data privacy challenges and increase receptivity to LLM deployment in the clinical community, future studies should also consider and evaluate open-source LLMs.

Thus, future developments must emphasize multimodal synergies (between LLMs and deep AI and ML), standardize development and testing, enhance explainability, and conduct deeper investigations into implementation and deployment practices that engage patients, centering on their well-being.

### Conclusions

In this scoping review, LLMs have demonstrated considerable potential to transform aspects of mental health care. However, current implementations remain predominantly experimental and qualify as preliminary proof-of-concept studies. While many studies reported superior performance of LLMs, these studies are characterized by risk of publication bias as well as heterogeneous study designs. Critically, only a minority of studies (n=13, 31.7%) [20,23,36,38,41,43,49,50,52,55-58] validated LLM performance against clinician assessments using real patient data, with the majority relying on proxy outcomes such as clinical vignettes, exam questions, or social media posts. This validation gap potentially overestimates clinical use and limits generalizability to real-world practice. Currently, model tuning is the predominant method for training LLMs toward specific tasks, but this needs proper standardization and guidelines to ensure repeatability and reproducibility. Moreover, common frameworks for model evaluation to ensure safety and efficacy must precede implementation. In future works, prioritizing patient well-being as the paramount principle must remain throughout technology development, testing, and operational deployment.

## Appendix

Multimedia Appendix 1 Specific search strategies and the full list of search terms.

Multimedia Appendix 2 PRISMA-ScR checklist.

## Abbreviations

AI: artificial intelligence
BERT: Bidirectional Encoder Representations from Transformers
CNN: convolutional neural network
DSM-5: Diagnostic and Statistical Manual of Mental Disorders, Fifth Edition
EHR: electronic health record
LLM: large language model
LSTM: long short-term memory
ML: machine learning
OCD: obsessive-compulsive disorder
PHQ-9: Patient Health Questionnaire-9
SVM: support vector machine
