# Supplementary material for: Large Language Models and Their Applications in Mental Health: Scoping Review
Source: JMIR Ment Health. 2026 May 15;13:e88057. doi: 10.2196/88057 (PMC13537432; doi:10.2196/88057)
Supplement: Supplementary file 1 [file mental_v13i1e88057_app1.docx]

## Search Terms (as of 1^st^ November 2025)

### PubMed

((((psychiatry[Title/Abstract]) OR (mental health[Title/Abstract])) AND ((LLM[Title/Abstract]) OR (large language model[Title/Abstract]) or (large language models[Title/Abstract]) or (LLMs[Title/Abstract]))) AND 2023/01/01:2025/10/31[dp]) AND (english[Language])

### World of Science

(TS=("mental health" OR "psychiatry") AND TS=("LLM" OR "large language model" or "large language models" or "LLMs")) OR (Ti=("mental health" OR "psychiatry ") AND Ti=("LLM" OR "large language model" or "large language models" or "LLMs")) OR (Ab=("mental health" OR "psychiatry ") AND Ab=("LLM" OR "large language model" or "large language models" or "LLMs")) AND DOP=(2023-01-01/2025-10-31)

Filter by Dcoument Types = Article and Language = English

### Scopus

TITLE-ABS-KEY("mental health" OR "psychiatry" OR "psychosis") AND TITLE-ABS-KEY("llm" OR "large language model" OR "gemini" OR "GPT" OR "ChatGPT" OR "llama") AND PUBYEAR AFT 2022 AND PUBYEAR BEF 2026

Article Type = Research articles, language = English
